# Curcumin, Alone or in Combination with Aminoguanidine, Increases Antioxidant Defenses and Glycation Product Detoxification in Streptozotocin-Diabetic Rats: A Therapeutic Strategy to Mitigate Glycoxidative Stress

**DOI:** 10.1155/2020/1036360

**Published:** 2020-05-20

**Authors:** Tayra Ferreira Oliveira Lima, Mariana Campos Costa, Ingrid Delbone Figueiredo, Maiara Destro Inácio, Maria Rita Rodrigues, Renata Pires Assis, Amanda Martins Baviera, Iguatemy Lourenço Brunetti

**Affiliations:** ^1^São Paulo State University (UNESP), School of Pharmaceutical Sciences, Department of Clinical Analysis, Araraquara, São Paulo, Brazil; ^2^Federal University of Alfenas (UNIFAL-MG), School of Pharmaceutical Sciences, Department of Clinical and Toxicological Analysis, Alfenas, Minas Gerais, Brazil; ^3^Paulista University (UNIP), Institute of Health Sciences, Araraquara, São Paulo, Brazil

## Abstract

Both oxidative stress and the exacerbated generation of advanced glycation end products (AGEs) have crucial roles in the onset and progression of diabetic complications. Curcumin has antioxidant and antidiabetic properties; its combination with compounds capable of preventing the advanced glycation events, such as aminoguanidine, is an interesting therapeutic option to counteract diabetic complications. This study is aimed at investigating the effects of treatments with curcumin or aminoguanidine, alone or in combination, on metabolic alterations in streptozotocin-diabetic rats; the focus was mainly on the potential of these bioactive compounds to oppose the glycoxidative stress. Curcumin (90 mg/kg) or aminoguanidine (50 and 100 mg/kg), alone or in combination, slightly decreased glycemia and the biomarkers of early protein glycation, but markedly decreased AGE levels (biomarkers of advanced glycation) and oxidative damage biomarkers in the plasma, liver, and kidney of diabetic rats. Some novel insights about the *in vivo* effects of these bioactive compounds are centered on the triggering of cytoprotective machinery. The treatments with curcumin and/or aminoguanidine increased the activities of the antioxidant enzymes (paraoxonase 1, superoxide dismutase, and catalase) and the levels of AGE detoxification system components (AGE-R1 receptor and glyoxalase 1). In addition, combination therapy between curcumin and aminoguanidine effectively prevented dyslipidemia in diabetic rats. These findings demonstrate the combination of curcumin (natural antioxidant) and aminoguanidine (prototype therapeutic agent with anti-AGE activity) as a potential complementary therapeutic option for use with antihyperglycemic agents, which may aggregate beneficial effects against diabetic complications.

## 1. Introduction

The increasing prevalence of diabetes mellitus (DM) is a major health concern worldwide. Longer duration of diabetes may directly influence the incidence of microvascular (kidney failure, retinopathy, neuropathy, and lower extremity amputations) and macrovascular (cardiovascular diseases) complications [1]. Diabetic complications have been associated with poor metabolic control and transient episodes of hyperglycemia, which result in the phenomenon called “metabolic memory” that causes relevant changes in many tissues. Metabolic memory is a term used to describe the persistence of the diabetic complications, even after the achievement of metabolic control; it is the collective result of several mechanisms, including the generation of advanced glycation products, oxidative stress, inflammation, and epigenetic changes [2]. From a clinical point of view, metabolic memory necessitates rapid intensive treatment of diabetic individuals following the diagnosis to quickly achieve metabolic control and minimize the long-term detrimental impacts of hyperglycemia in tissues [3, 4].

In addition to an intensive and more prompt therapy for glycemic control in diabetic individuals, novel combined therapeutic approaches have been suggested, including the use of bioactive agents capable of inhibiting the biochemical cascades triggered by advanced glycation, reactive oxygen species (ROS), and inflammation, thus effectively mitigating diabetic complications [5].

In this context, combined therapies based on natural bioactive compounds with multiple effects against both symptoms and complications of diseases are currently an emerging trend. Many studies have reported that combinations of certain natural bioactive substances have beneficial effects on diabetes, particularly owing to their antioxidant properties [6, 7]. Additionally, strategies of combined therapies of natural bioactive compounds and antidiabetic drugs, including their combinations with insulin [8, 9] or metformin [10–12], have been proposed recently in an attempt to improve the glycemic control, decrease dyslipidemia, and mitigate the diabetic complications related to oxidative stress. Curcumin (diferuloylmethane; C_21_H_20_O_6_) has gained attention as an interesting candidate for combined therapies aimed at DM management, considering the large amount of evidence from preclinical and clinical studies demonstrating its antihyperglycemic, anti-inflammatory, and antioxidant activities, which have been useful to attenuate diabetic complications [13, 14]. Nevertheless, there is the need for more studies on natural bioactive compounds to investigate their potentials in mitigating the advanced glycation events, alone or in combined therapy approaches.

The formation of advanced glycation end products (AGEs) is accelerated under conditions of hyperglycemia. Protein glycation and the modification of amino acid residues by dicarbonyl compounds, including glyoxal, methylglyoxal, and 3-deoxyglucosone, are the main precursors of AGEs [15]. AGEs contribute to the onset of diabetic complications, mostly via two mechanisms: (i) formation of crosslink in biomolecules, thus altering their structure and function, and (ii) interacting with the RAGE receptor on cell surfaces, thus stimulating signaling pathways that lead to oxidative stress exacerbation [16]. Therefore, it is reasonable to propose that, in addition to dampening oxidative stress, some natural bioactive compounds are also able to inhibit the deleterious impacts of AGEs; this apparently makes them interesting candidates for strategies of combined therapies against diabetic disturbances. Bioactive substances with anti-AGE properties can act through the inhibition of AGEs formation, due to their intrinsic ability to scavenge protein glycation intermediates and/or dicarbonyl compounds. For example, aminoguanidine (pimagedine, CH_6_N_4_), a prototype therapeutic agent, reacts with methylglyoxal. Moreover, anti-AGE agents may also stimulate the endogenous systems involved in AGE detoxification, which include the AGE-receptor family (AGE-R) and the glyoxalase system. AGE-R consists of three receptors, including the AGE-R1 (OST-48) receptor. AGE-R1 is involved in the endocytic uptake and degradation of proteins modified by AGEs [17]. The glyoxalase system is involved in the detoxification of methylglyoxal, which is metabolized by the glyoxalase 1 (GLO 1) to S-_D_-lactoylglutathione, which is then transformed into _D_-lactate by the glyoxalase 2 (GLO 2) [18].

The aim of this study was to investigate whether treatments based on curcumin and aminoguanidine, individually or in combination, have beneficial effects on the metabolic disturbances observed in streptozotocin-induced diabetic rats. The ability of these bioactive compounds to stimulate the endogenous antioxidant defenses and the components involved in AGEs detoxification was the main focus of this study. The results of this investigation could present initial evidence for a new therapeutic strategy to counteract the diabetic complications related to glycoxidative stress, as the option could become complementary to more classical therapies based on antidiabetic agents.

## 2. Materials and Methods

### 2.1. Experimental Animals

Male Wistar rats (*Rattus norvegicus*) weighing 140-160 g (6 weeks old) were maintained under controlled conditions (23 ± 1°C, humidity 55% ± 5%, 12 h light/12 h dark cycle) on a lab chow diet (Presence, Brazil) and with water always available. Before initiating the experiments, all animals had a 3-day adaptation period. The animal experiments were carried out with the approval of the Committee for Ethics in Animal Experimentation from the School of Pharmaceutical Sciences, UNESP, Araraquara (CEUA/FCF/CAr resolution number 25/2016).

### 2.2. Induction of Diabetes Mellitus and Experimental Design

The rats were fasted for 12 hours and then received an intravenous injection of streptozotocin (STZ) proportional to their body weight (40 mg/kg, Sigma-Aldrich, St. Louis, Missouri, USA) prepared in 0.01 M citrate buffer (pH 4.5) to induce the experimental type 1 DM. The rats intended for the control group, nondiabetic rats, only received citrate buffer. For this procedure, all the rats were previously anesthetized by isoflurane inhalation. Three days after STZ administration, postprandial glycemia was measured by the glucose oxidase method using a commercial kit (Labtest Diagnostica SA, Lagoa Santa, Minas Gerais, Brazil) and used to compose the experimental groups by matching specimens with similar values of glycemia and body weight. This constituted “day 0” of the experiment, after which began the animal treatment with insulin, curcumin, or aminoguanidine.

Diabetic rats with postprandial glycemia concentrations of approximately 400 mg/dL were divided into the different experimental groups (10 rats/group), as follows: diabetic rats treated with (i) yoghurt (DYOG); (ii) 90 mg/kg curcumin in yoghurt (DC); (iii) 50 mg/kg aminoguanidine in yoghurt (DA-50); (iv) 100 mg/kg aminoguanidine in yoghurt (DA-100); (v) 90 mg/kg curcumin+50 mg/kg aminoguanidine in yoghurt (DCA-50); (vi) 90 mg/kg curcumin+100 mg/kg aminoguanidine in yoghurt (DCA-100); and (vii) 4 U/day insulin (DINS). Normal rats treated with yoghurt (NYOG group) (viii) were also kept as controls.

Curcumin (from *Curcuma longa* L.; 78%, Sigma-Aldrich, St. Louis, Missouri, USA, C1386, batch number SLBN7214V) and aminoguanidine (97%, Sigma-Aldrich, St. Louis, Missouri, USA, 109266, batch number STBD3851V) were mixed (alone or in combination) with commercial plain yoghurt (Nestlé®) as described by Assis et al. [7]. All the treatments were administered, by half doses twice a day, at 08:00 and 17:00, for 45 days. Curcumin and/or aminoguanidine were administered by gavage (45 mg/kg for curcumin, 25 mg/kg and 50 mg/kg for minor and major aminoguanidine doses, respectively) in 0.5 mL of yoghurt (totalizing 1.0 mL/day). Insulin was given twice a day, as subcutaneous injections (2 U of insulin per injection, Biohulin®, NU-100, Brazil).

During the experimental period, body weight and glycemia were assessed every 15 days. Glucose levels were determined in plasma obtained from blood samples collected from the tip of the tail in heparinized tubes (Hemofol®, 5,000 IU/mL, Brazil). On the 40^th^ treatment day, the animals were housed into individual metabolic cages on an *ad libitum* water and lab chow diet for adaptation. For the whole 24 h of the 43^rd^ day, urine samples were collected to measure urinary protein levels; the food intake was also monitored. Plasma glucose levels and urinary protein levels were determined using commercial kits (Labtest Diagnostica SA, Lagoa Santa, Minas Gerais, Brazil).

At the end of the treatments, the activities of alanine aminotransferase (ALT), aspartate aminotransferase (AST), and alkaline phosphatase (ALP) were determined in plasma obtained from the blood samples collected from the tip of the tail. After that, rats were euthanized by decapitation and blood samples were collected for the analysis of glycated hemoglobin (HbA1c); for the plasma levels of glucose, fructosamine, triglycerides, and total cholesterol (Labtest Diagnostica SA, Lagoa Santa, Minas Gerais, Brazil); for the measurement of biomarkers of glycoxidative damage [thiobarbituric acid reactive substances (TBARS), protein carbonyl groups (PCO), fluorescent AGEs, and total nitrate and nitrite (NO_x_)] and of the activities of antioxidant enzyme paraoxonase 1 (PON 1). The liver and kidneys were removed, snap-frozen in liquid nitrogen, and stored at -80°C for the analysis of biomarkers of glycoxidative damage (TBARS, PCO, fluorescent AGEs), antioxidant defenses [superoxide dismutase (SOD), catalase (CAT), glutathione peroxidase (GSH-Px), glutathione reductase (GSH-Rd), and nonprotein sulfhydryl (NPSH) groups], and components belonging to AGE detoxification systems (AGE-R1 receptor and GLO 1).

### 2.3. Determination of Antioxidant Enzymes and Metabolites

PON 1 plasma activity was determined as in Assis et al. [7], by monitoring the *p*-nitrophenol (405 nm) released from the hydrolysis of paraoxon.

The activities of the antioxidant enzymes SOD, CAT, GSH-Px, and GSH-Rd were monitored in the liver and kidneys, using supernatants of tissue homogenates. The oxidation of xanthine by xanthine oxidase generates the superoxide anion radical, which reduces the nitroblue tetrazolium chloride (NBT); the SOD catalyzes the dismutation of superoxide anion radicals and inhibits NBT reduction. SOD activity was assessed by monitoring the inhibition of NBT reduction at 550 nm [19]. CAT activity was assessed by measuring the decrease in the absorbance at 230 nm, due to the consumption of hydrogen peroxide (H_2_O_2_) [20]. The activities of GSH-Px and GSH-Rd were measured according to Rush and Sandiford [21] and Carlberg and Mannervik [22], respectively, monitoring the oxidation of NADPH to NADP^+^ (340 nm), since it is concomitant with the reduction of oxidized glutathione. The protein levels in tissue supernatants were determined according to Lowry et al. [23] to provide a corrective parameter for results related to SOD, CAT, GSH-Px, and GSH-Rd activities.

Liver and kidney levels of NPSH groups (which represent an indirect measurement of the reduced glutathione, GSH) were determined by the reduction of 5,5-dithiobis-(2-nitrobenzoic acid) at 412 nm [24].

### 2.4. Determination of Glycoxidative Stress Biomarkers

The levels of fluorescent AGEs (biomarkers of advanced glycation), PCO (biomarker of protein oxidation), and TBARS (biomarkers of lipid peroxidation, LPO) were determined in plasma and in the homogenates of the liver and kidneys. LPO products were evaluated by the thiobarbituric acid (TBA) test [25]; the assay is based on TBA reactivity mainly with malondialdehyde, which generates products (TBARS) whose levels were measured spectrophotometrically (535 nm) in the liver and kidneys or spectrofluorometrically (excitation and emission wavelengths of 510 nm and 553 nm, respectively) in plasma. PCO levels were determined by monitoring the generation of 2,4-dinitrophenylhydrazone (370 nm) after the reaction of the carbonyl groups in proteins with 2,4-dinitrophenylhydrazine [26]. The estimation of fluorescent AGEs was made spectrofluorometrically (excitation and emission wavelengths of 370 nm and 440 nm, respectively) as described by Costa et al. [27]. The protein levels in plasma and tissue supernatants were determined according to Lowry et al. [23] for the correction of the results related to fluorescent AGEs and PCO.

The estimation of plasma nitric oxide (NO) levels was performed indirectly according to Miranda et al. [28], with modifications, by quantifying its stable oxidation pathways' final products, nitrate and nitrite. Plasma NO_x_ levels, which indirectly indicate the activity of inducible nitric oxide synthase (iNOS), were determined using the Griess reagent and reducing nitrous species with vanadium (III) chloride (VCl_3_). Plasma was deproteinized with 70 mM zinc acetate following by centrifugation at 10,000 *g* for 15 min at 4°C. Supernatants were incubated with 17 mM VCl_3_ and Griess reagent for 30 min at 37°C. The tubes were centrifuged at 10,000 *g* for 2 min at 25°C. NO_x_ levels in supernatants were monitored spectrophotometrically (540 nm). Nitrate (Sigma-Aldrich, St. Louis, Missouri, USA) was used as a standard.

### 2.5. Western Blot Analysis

The liver and kidney samples were prepared as described by Costa et al. [27]. Samples containing 30-50 *μ*g of proteins were subjected to SDS-PAGE electrophoresis on 10-12% acrylamide gels and were then electroblotted onto nitrocellulose membranes. Proteins were detected by overnight incubation at 4°C with specific primary antibodies: anti-AGE-R1 (anti-DDOST) (1 : 500) and anti-GLO 1 (1 : 1000) (Abcam, Cambridge, UK). Anti-*β*-actin (1 : 500; Cell Signaling, Danvers, MA) was used as an internal control. Primary antibody binding was detected by peroxidase-conjugated secondary antibodies (anti-rabbit IgG, HRP-linked antibody (1 : 1000) or anti-mouse IgG, HRP-linked antibody (1 : 2000); Cell Signaling, Danvers, MA) and visualized using an enhanced chemiluminescence substrate. Chemiluminescent bands were captured, and intensities were analyzed [27]. All results were normalized to *β*-actin and expressed as a percentage compared to the NYOG group.

### 2.6. Statistical Analysis

Data were expressed as the mean ± standard error of mean (SEM). One-way analysis of variance (ANOVA) followed by the Student-Newman-Keuls test was used to compare the intergroup differences. Paired Student's *t*-test was used to compare the intragroup changes, relative to day 0. Data were considered statistically different at *p* < 0.05. Statistical analyses were performed using the program GraphPad Prism 6.01 (GraphPad Software, San Diego, California, USA).

## 3. Results

### 3.1. Body Weight and Food Intake

**Figure 1:**
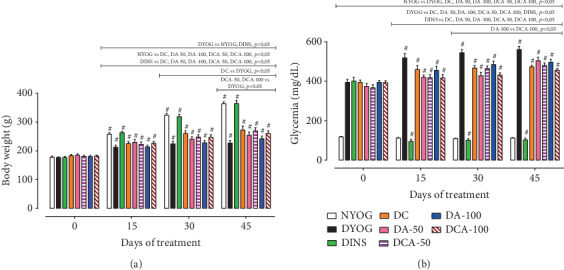
Body weight (a) and glycemia levels (b) of streptozotocin-induced diabetic rats treated for 45 days with yoghurt enriched with curcumin, alone or in combination with aminoguanidine. Values are expressed as the mean ± standard error of the mean (SEM) (*n* = 10). Differences between groups were considered significant at *p* < 0.05 and were analyzed using one-way ANOVA followed by the Student-Newman-Keuls test. Differences in the same group relative to day 0 were analyzed using paired Student's *t*-test. ^#^Different compared to day 0 (*p* < 0.05). Here and in Figures 2to 8—NYOG: normal rats treated with yoghurt; DYOG: diabetic rats treated with yoghurt; DINS: diabetic rats treated with 4 U/day insulin; DC: diabetic rats treated with 90 mg/kg curcumin in yoghurt; DA-50: diabetic rats treated with 50 mg/kg aminoguanidine in yoghurt; DCA-50: diabetic rats treated with 90 mg/kg curcumin+50 mg/kg aminoguanidine in yoghurt; DA-100: diabetic rats treated with 100 mg/kg aminoguanidine in yoghurt; DCA-100: diabetic rats treated with 90 mg/kg curcumin+100 mg/kg aminoguanidine in yoghurt.

In agreement with the catabolic state observed in rats under a model of experimental type 1 DM, DYOG rats had lower body weight throughout the experimental period than NYOG rats. As expected, DINS rats had increased body weight gain, similarly to NYOG rats (Figure 1(a)).

The DC group showed a slight increase in the body weight gain from the 30^th^ day of treatment; however, it remained lower than in DINS and NYOG rats (Figure 1(a)). Groups DA-50 and DA-100 had no improvement of body weight gain (Figure 1(a)).

After 45 days, the DCA-50 and DCA-100 groups had slight body weight gain increases (Figure 1(a)) similar to what was observed after the treatment with curcumin alone.

After 43 days, in addition to low body weight gain, diabetic rats also had polyphagia, a classic symptom of this experimental type 1 DM model; the food intake of the DYOG group was higher (49.05 ± 2.75 g/24 h) than in the NYOG group (24.9 ± 1.40 g/24 h). As expected, the treatment of diabetic rats with insulin decreased the food intake (32.35 ± 2.05 g/24 h), which still remained higher than in NYOG rats.

The food intake had a slight decrease in DC rats (40.78 ± 0.92 g/24 h) but was still higher than in the DINS and NYOG groups. On the other hand, DA-50 (43.95 ± 1.15 g/24 h) and DA-100 (45.65 ± 1.45 g/24 h) did not show changes of food intake.

Both combined treatments of curcumin+50 mg/kg aminoguanidine (39.90 ± 0.60 g/24 h) and curcumin+100 mg/kg aminoguanidine (40.55 ± 1.55 g/24 h) caused slight decreases in the food intake, similarly to the effects of curcumin alone.

### 3.2. Glycemia

All diabetic animal groups started the experiment with blood glucose levels of approximately 400 mg/dL (Figure 1(b)). After 15 days, DYOG rats showed significant increases in the glycemia levels, which remained high until the end of the experimental period, evidencing the worsening of DM. As expected, diabetic rats treated with insulin had reductions in the glycemia, whose levels were similar to those of NYOG rats. Glycemia of NYOG rats remained within normal values until the end of the experiment (Figure 1(b)). The treatment of diabetic rats with curcumin or with aminoguanidine at both doses, alone, decreased the glycemia levels, although with less efficiency in relation to the treatment with insulin (Figure 1(b)).

The combined therapies of curcumin+aminoguanidine at both doses decreased the glycemia of diabetic rats. The DCA-50 group preserved the beneficial effects of the isolated treatments, whereas the DCA-100 group showed the cumulated benefits from the aminoguanidine treatment, since the glycemia levels of DCA-100 rats were minor than those of DA-100 rats, which represents the maintenance of the curcumin effects (Figure 1(b)).

### 3.3. HbA1c and Fructosamine

To verify the correlations between the changes in the glycemia and the levels of biomarkers related to protein glycation in the different groups, the levels of HbA1c and fructosamine were evaluated. Both HbA1c and fructosamine are Amadori products (ketoamine products with relative stability) irreversibly formed during the early steps of nonenzymatic protein glycation; their levels are positively correlated with blood glucose level fluctuations [29].

After 45 days of experiment, DYOG rats had high levels of both HbA1c and fructosamine, in line with the high levels of plasma glucose and the worsening of DM. Among all treated groups, DINS rats had the lowest levels of HbA1c and fructosamine, still higher than NYOG rats (Table 1).

The treatments with curcumin or aminoguanidine at both doses, alone or in combination, decreased the HbA1c and fructosamine levels of diabetic rats but did not reach the efficiency of insulin or inhibit the early steps of protein glycation (Table 1).

### 3.4. Lipid Profile

After 45 days of experiment, dyslipidemia was observed in DYOG rats, since they had increased plasma levels of triglycerides and total cholesterol. Insulin treatment prevented dyslipidemia, since the triglycerides and total cholesterol levels in the plasma of DINS rats were similar to those of NYOG rats (Table 1).

Both triglycerides and total cholesterol decreased in the plasma of diabetic rats treated with curcumin or aminoguanidine (50 and 100 mg/kg) alone but remained still higher than in DINS and NYOG rats (Table 1).

The combined therapy of curcumin+aminoguanidine added benefits to the control of plasma lipid levels of diabetic rats. The treatments with curcumin+50 mg/kg aminoguanidine and with curcumin+100 mg/kg aminoguanidine effectively decreased dyslipidemia of diabetic rats, as they maintained plasma triglycerides and total cholesterol at levels similar to those of DINS and NYOG rats (Table 1).

### 3.5. Biomarkers of Hepatic Damage

After 45 days of experiment, the levels of biomarkers related to hepatocyte damage and hepatobiliary pancreatitis (ALT, AST, and ALP) were increased in the plasma of DYOG rats (Table 1). The treatment of diabetic rats with insulin best attenuated the hepatic damage, since the plasma levels of ALT, AST, and ALP were significantly decreased when compared to values of DYOG rats, with their levels being similar to those of NYOG rats (Table 1).

The treatments with curcumin or with 50 mg/kg aminoguanidine, alone or in combination, decreased the plasma levels of ALT, AST, and ALP (Table 1).

Both treatments with 100 mg/kg aminoguanidine, alone or in combination with curcumin, significantly reduced the levels of ALT and AST; however, these treatments did not avoid the increase of the ALP levels (Table 1), suggesting that the high dose of aminoguanidine, alone or coadministered with curcumin, could be ineffective on hepatobiliary pancreatitis or even toxic for the hepatobiliary system of STZ-diabetic rats.

### 3.6. Biomarker of Renal Damage

DYOG rats showed increases in the protein urinary levels, which suggested the onset of kidney damage. DINS rats had proteinuria levels similar to NYOG rats, lower than in the DYOG group (Table 1).

The treatment with 90 mg/kg curcumin alone decreased the proteinuria levels of diabetic rats. In contrast, treatments with aminoguanidine alone, at both doses, were not able to prevent the proteinuria in diabetic rats (Table 1).

The treatment with curcumin+50 mg/kg aminoguanidine decreased the proteinuria levels of diabetic rats, similar to the effect of curcumin alone. On the other hand, the treatment with curcumin+100 mg/kg aminoguanidine was not able to decrease the proteinuria, whose levels were similar to those of diabetic rats treated with 100 mg/kg aminoguanidine alone (Table 1).

### 3.7. Biomarkers of Glycoxidative Damage and Antioxidant Defenses in Plasma

**Figure 2:**
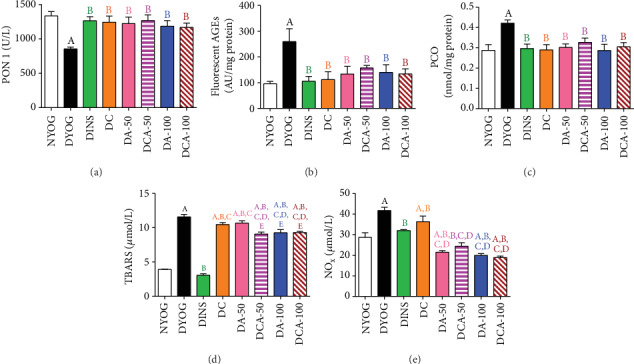
Antioxidant defenses and glycoxidative stress biomarkers in the plasma of streptozotocin-induced diabetic rats after 45 days of treatment with yoghurt enriched with curcumin, alone or in combination with aminoguanidine. Activity of PON 1 (a) and levels of fluorescent AGEs (b), PCO (c), TBARS (d), and NO_x_ (e). Values are expressed as the mean ± standard error of the mean (SEM) (*n* = 10). Differences between groups were considered significant at *p* < 0.05 and were analyzed using one-way ANOVA followed by the Student-Newman-Keuls test. ^A^Differences to NYOG. ^B^Differences to DYOG. ^C^Differences to DINS. ^D^Differences to DC. ^E^Differences to DA-50.

In the plasma of DYOG rats, the PON 1 activity was decreased (Figure 2(a)), whereas the levels of fluorescent AGEs (Figure 2(b)), PCO (Figure 2(c)), TBARS (Figure 2(d)), and NO_x_ (Figure 2(e)) were significantly increased, in accordance with the onset of glycoxidative stress in STZ-induced diabetic rats. Insulin treatment prevented the onset of glycoxidative stress in diabetic rats.

Curcumin and aminoguanidine at both doses, alone or in combination, increased PON 1 activity and reduced the plasma levels of fluorescent AGEs (Figure 2(b)) and PCO (Figure 2(c)) to values similar to those of DINS and NYOG rats. These findings suggest that these bioactive compounds are beneficial for dampening the glycoxidative stress in diabetes.

Diabetic rats treated with curcumin or aminoguanidine alone had lower TBARS levels than DYOG rats; in DA-100 rats, TBARS plasma levels were even lower than in DA-50 and DC rats (Figure 2(d)). The combined treatment of curcumin+50 mg/kg aminoguanidine was effective against lipid peroxidation, since TBARS plasma levels were further decreased when compared to the treatments with curcumin or aminoguanidine alone. The combined treatment of curcumin+100 mg/kg aminoguanidine was also effective in mitigating lipid peroxidation, since it maintained the aminoguanidine effect on reducing the TBARS plasma levels in diabetic rats (Figure 2(d)).

NO_x_ plasma levels decreased in the DINS group. Aminoguanidine alone, at both doses, significantly reduced plasma NO_x_ levels to values lower than those in NYOG and DINS rats. On the other hand, treatment with 90 mg/kg curcumin alone slightly decreased the plasma levels of NO_x_. Diabetic rats treated with the combined treatment of curcumin+aminoguanidine, at both doses, aggregated benefits to curcumin, since the plasma NO_x_ levels were significantly reduced in the DCA-50 and DCA-100 groups, when compared to DYOG rats (Figure 2(e)).

### 3.8. Antioxidant Defenses and AGE Detoxification Components in the Liver and Kidney

Similar to the impairments observed for PON 1, the activities of other important enzymes involved in ROS detoxification, as well as the NPSH levels and the components related to AGE detoxification, were altered in STZ-diabetic rats.

**Figure 3:**
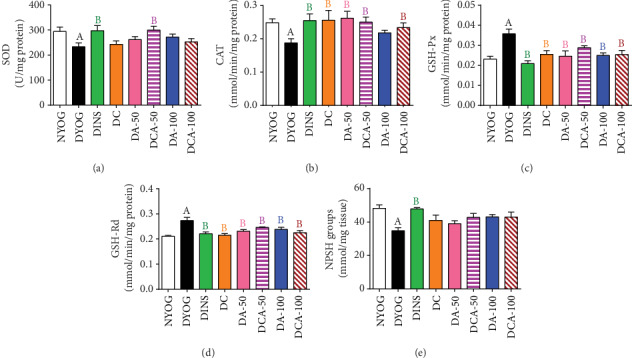
Antioxidant system biomarkers in the liver of streptozotocin-induced diabetic rats after 45 days of treatment with yoghurt enriched with curcumin, alone or in combination with aminoguanidine. Activities of the enzymes SOD (a), CAT (b), GSH-Px (c), and GSH-Rd (d) and levels of NPSH groups (e). Values are expressed as the mean ± standard error of the mean (SEM) (*n* = 10). Differences between groups were considered significant at *p* < 0.05 and were analyzed using one-way ANOVA followed by the Student-Newman-Keuls test. ^A^Differences to NYOG. ^B^Differences to DYOG.

**Figure 4:**
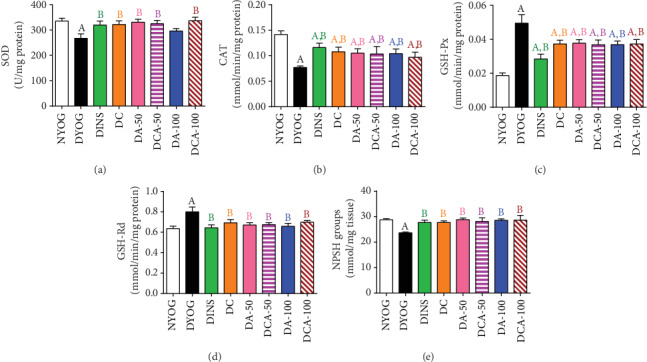
Antioxidant system biomarkers in the kidney of streptozotocin-induced diabetic rats after 45 days of treatment with yoghurt enriched with curcumin, alone or in combination with aminoguanidine. Activities of SOD (a), CAT (b), GSH-Px (c), and GSH-Rd (d) and levels of NPSH groups (e). Values are expressed as the mean ± standard error of the mean (SEM) (*n* = 10). Differences between groups were considered significant at *p* < 0.05 and were analyzed using one-way ANOVA followed by the Student-Newman-Keuls test. ^A^Differences to NYOG. ^B^Differences to DYOG.

**Figure 5:**
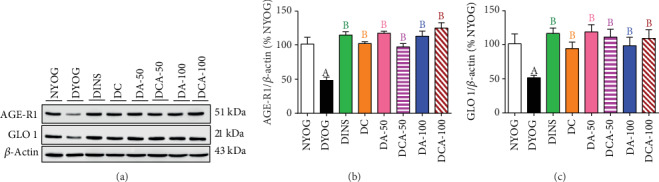
AGE detoxification biomarkers in the liver of streptozotocin-induced diabetic rats after 45 days of treatment with yoghurt enriched with curcumin, alone or in combination with aminoguanidine. Representative lanes of protein levels as detected by western blot analysis for AGE-R1, GLO 1, and *β*-actin (a); densitometric analysis of western blot results for AGE-R1 (b) and GLO 1 (c). Values are expressed as the mean ± standard error of the mean (SEM) (*n* = 6). Differences between groups were considered significant at *p* < 0.05 and were analyzed using one-way ANOVA followed by the Student-Newman-Keuls test. ^A^Differences to NYOG. ^B^Differences to DYOG.

**Figure 6:**
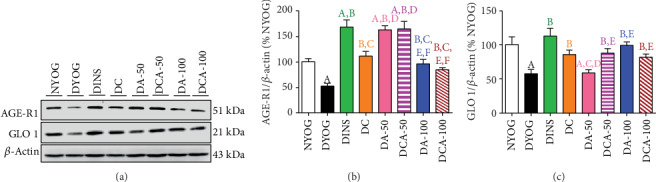
AGE detoxification biomarkers in the kidney of streptozotocin-induced diabetic rats after 45 days of treatment with yoghurt enriched with curcumin, alone or in combination with aminoguanidine. Representative lanes of protein levels as detected by western blot analysis for AGE-R1, GLO 1, and *β*-actin (a); densitometric analysis of western blot results for AGE-R1 (b) and GLO 1 (c). Values are expressed as the mean ± standard error of the mean (SEM) (*n* = 6). Differences between groups were considered significant at *p* < 0.05 and were analyzed using one-way ANOVA followed by the Student-Newman-Keuls test. ^A^Differences to NYOG. ^B^Differences to DYOG. ^C^Differences to DINS. ^D^Differences to DC. ^E^Differences to DA-50. ^F^Differences to DCA-50.

DYOG rats had significant decreases in various cytoprotective components in both the liver and kidneys. The activities of the antioxidant enzymes SOD (liver and kidney, in Figures 3(a) and 4(a), respectively) and CAT (liver and kidney, in Figures 3(b) and 4(b), respectively), as well as the levels of NPSH (liver and kidney, in Figures 3(e) and 4(e), respectively) decreased. The components of AGE detoxification system, including AGE-R1 (liver and kidney, in Figures 5(b) and 6(b), respectively) and GLO 1 (liver and kidney, in Figures 5(c) and 6(c), respectively), were significantly lower in DYOG rats. Interestingly, DYOG rats had increased activities of GSH-Px and GSH-Rd in the liver (Figures 3(c) and 3(d), respectively) and kidney (Figures 4(c) and 4(d), respectively), when compared with NYOG rats; these changes in the components of the glutathione redox system may be interpreted as a compensatory mechanism against oxidative stress. The treatment of diabetic rats with insulin prevented all these alterations.

The treatments of diabetic rats with curcumin or with 50 mg/kg aminoguanidine, alone, increased the SOD activity in the kidney (Figure 4(a)), but not in the liver (Figure 3(a)). The activities of CAT in the liver (Figure 3(b)) and kidney (Figure 4(b)) of diabetic rats were enhanced by the treatments with curcumin alone or 50 mg/kg aminoguanidine alone.

However, a distinct response was achieved when curcumin and 50 mg/kg aminoguanidine were administered in combination to diabetic rats, showing a better response in the antioxidant defenses. The benefits previously achieved with the single compound therapies were maintained (i.e., the increases in the activities of SOD and CAT in the kidneys and the increase of hepatic CAT activity). Additionally, curcumin+50 mg/kg aminoguanidine promoted a significant increase in hepatic SOD activity (Figure 3(a)), showing the potential of this combined therapy in combating oxidative stress in diabetes.

The treatment with 100 mg/kg aminoguanidine alone was less effective than the lower dose in maintaining the antioxidant responses in the liver and kidneys. Although 100 mg/kg aminoguanidine increased the renal CAT activity (Figure 4(b)), it was not effective in increasing the activity of this enzyme in the liver (Figure 3(b)), nor did it improve SOD activity in both tissues (Figures 3(a) and 4(a)). The combined therapy added benefits to aminoguanidine efficacy in recovering the antioxidant defenses. Curcumin+100 mg/kg aminoguanidine increased the activities of CAT in the liver and kidney (Figures 3(b) and 4(b), respectively) and the activity of SOD in the kidney (Figure 4(a)), showing cumulative beneficial effects; however, this combined therapy was unable to improve SOD activity in the liver (Figure 3(a)).

The activities of GSH-Px and GSH-Rd in the liver (Figures 3(c) and 3(d), respectively) and kidney (Figures 4(c) and 4(d), respectively) were similar among all treatments and had the same values as NYOG rats, except for GSH-Px in the kidneys that had lower activity than in DYOG rats, but still higher than in the NYOG rats. The levels of NPSH in the kidneys of diabetic rats increased similarly after all treatments (Figure 4(e)); on the other hand, insulin was the unique treatment capable of recovering hepatic NPSH levels (Figure 3(e)).

In the liver of diabetic rats, the treatments with curcumin or aminoguanidine, alone or in combination, significantly increased the protein levels of AGE-R1 (Figures 5(a) and 5(b)) and GLO 1 (Figures 5(a) and 5(c)). The benefits of these treatments were similar to those of insulin therapy, increasing the AGE-R1 and GLO 1 to levels similar to those in NYOG rats.

In the kidney of diabetic rats, the highest increases in protein levels of AGE-R1 (Figure 6(b)) and GLO 1 (Figure 6(c)) were achieved through the insulin treatment. On the other hand, discrete effects on increasing AGE-R1 (Figure 6(b)) and GLO 1 (Figure 6(c)) levels were observed after treatment with curcumin alone. Treatments with aminoguanidine alone had interesting impacts on AGE detoxification components in the kidneys: 50 mg/kg aminoguanidine caused a robust increase in AGE-R1 levels (similar to the effects of insulin treatment) (Figure 6(b)) and practically did not improve GLO 1 levels (Figure 6(c)), whereas 100 mg/kg aminoguanidine caused a discrete increase in AGE-R1 (Figure 6(b)) and significantly increased the levels of GLO 1 (Figure 6(c)). Analyzing the effects of combined therapies and their impact on AGE detoxification components, curcumin+50 mg/kg aminoguanidine showed the best results, since this combination preserved the beneficial effects of both treatments, i.e., the increases in the levels of AGE-R1 (aminoguanidine effect; Figure 6(b)) and GLO 1 (curcumin effect; Figure 6(c)) in the kidneys. Curcumin+100 mg/kg aminoguanidine caused a slight increase in the renal levels of AGE-R1 (Figure 6(b)) and GLO 1 (Figure 6(c)).

### 3.9. Biomarkers of Glycoxidative Damage in the Liver and Kidney

**Figure 7:**
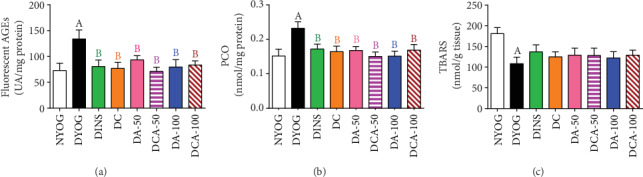
Glycoxidative stress biomarkers in the liver of streptozotocin-induced diabetic rats after 45 days of treatment with yoghurt enriched with curcumin, alone or in combination with aminoguanidine. Levels of fluorescent AGEs (a), PCO (b), and TBARS (c). Values are expressed as the mean ± standard error of the mean (SEM) (*n* = 10). Differences between groups were considered significant at *p* < 0.05 and were analyzed using one-way ANOVA followed by the Student-Newman-Keuls test. ^A^Differences to NYOG. ^B^Differences to DYOG.

**Figure 8:**
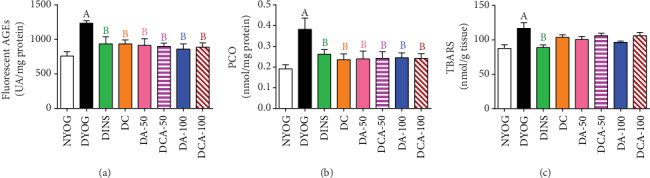
Glycoxidative stress biomarkers in the kidney of streptozotocin-induced diabetic rats after 45 days of treatment with yoghurt enriched with curcumin, alone or in combination with aminoguanidine. Levels of fluorescent AGEs (a), PCO (b), and TBARS (c). Values are expressed as the mean ± standard error of the mean (SEM) (*n* = 10). Differences between groups were considered significant at *p* < 0.05 and were analyzed using one-way ANOVA followed by the Student-Newman-Keuls test. ^A^Differences to NYOG. ^B^Differences to DYOG.

In addition to the detrimental impacts that occurred in plasma, glycoxidative stress was also observed in the liver and kidneys of DYOG rats, as increases were observed in the levels of fluorescent AGEs (as shown in Figures 7(a) and 8(a), respectively), PCO (Figures 7(b) and 8(b), respectively), and TBARS (kidney, Figure 8(c)). Interestingly, DYOG rats had reduced levels of TBARS in the liver (Figure 7(c)) in comparison with NYOG rats. As in plasma, the treatment with insulin prevented the onset of glycoxidative stress in the liver and kidney of diabetic rats.

Although the TBARS levels did not change in the liver (Figure 7(c)) and kidneys (Figure 8(c)) of diabetic rats treated with curcumin and aminoguanidine at both doses, alone or in combination, these treatments caused significant reductions in the levels of fluorescent AGEs and PCO in both the liver (Figures 7(a) and 7(b), respectively) and kidneys (Figures 8(a) and 8(b), respectively); these values being similar to those of DINS and NYOG rats, which reinforce the beneficial effects of these treatments on dampening the glycoxidative stress in diabetic rats.

## 4. Discussion

In the present study, we investigated the effects of single and combined therapies of curcumin and aminoguanidine on the physiological, biochemical, and glycoxidative stress parameters of STZ-diabetic rats. Furthermore, an important contribution of this study was the triggering of cytoprotective machinery involved in the elimination of ROS and products/intermediates of advanced glycation by curcumin and aminoguanidine treatments, which go beyond their well-known *per se* antioxidant (curcumin) and anti-AGE (aminoguanidine) properties. In this regard, when administered to diabetic rats alone or in combination, curcumin and aminoguanidine increased the endogenous antioxidant defenses, particularly the activities of PON 1 (plasma), SOD (liver and kidneys, mainly in combination), and CAT (liver and kidneys, alone or in combination); they also increased the expression of AGE detoxification components, including AGE-R1 and GLO 1 (liver and kidneys). The vast majority of the beneficial effects of combining curcumin and aminoguanidine have also been observed with the isolated compounds, especially the activation of antioxidant enzymes and AGE detoxification components. This can be interpreted as a positive result of the combination between these bioactive compounds, since their beneficial effects were not nullified when administered in combination. Furthermore, some additive effects were achieved with the combination of curcumin and aminoguanidine, especially the marked decrease in the levels of triglycerides, total cholesterol, and TBARS in the plasma of diabetic rats. Therefore, the combination of curcumin and aminoguanidine appears to be an interesting therapeutic strategy for the management of DM and prevention of the long-term complications of this disease.

In this study, normal (NYOG) and diabetic rats treated with yoghurt (DYOG) were considered the respective control groups for the absence or presence of metabolic disturbances and glycoxidative stress due to insulin deficiency. Treatment with yoghurt alone does not change the physiological and biochemical parameters classically altered in STZ-induced diabetic rats [30]. For this reason, studies in our laboratory have chosen yoghurt as the vehicle for oral administration of compounds that have low solubilities in water, such as curcumin, carotenoids, and other natural antioxidants [7, 27, 30]. Furthermore, the administration of phytochemicals or even antidiabetic drugs into yoghurt [10] did not impair their beneficial actions against the diabetic symptoms.

A group of diabetic rats treated with 4 U/day insulin (DINS) is often employed as a control group representing an effective pharmacological intervention for glycemia management in STZ-diabetic rats (characterized by severe insulinopenia); consequently, insulin administration prevents the diabetic disturbances related to glycoxidative stress. In fact, throughout the experimental period, the best effect on the control of plasma glucose levels was promoted by the treatment with 4 U/day insulin; treatments with curcumin, aminoguanidine, or their combinations did not achieve the same efficacy as insulin in glycemia management. This effective glycemic control probably explains the excellent effects of insulin on decreasing HbA1c and fructosamine levels, in comparison to the slight decreases promoted by curcumin or aminoguanidine, alone or in combination. It is well known that aminoguanidine acts by trapping or scavenging reactive carbonyl intermediates generated by the Maillard reaction, mainly hydroxyaldehydes and dicarbonyl compounds, including methylglyoxal [31]. Furthermore, it has been observed that curcumin also traps methylglyoxal [32]. Both HbA1c and fructosamine are Amadori products (ketoamine products with relative stability) formed in the early steps of protein glycation; curcumin and aminoguanidine inhibit the later stages of glycation. Therefore, it can be argued that the slight decreases observed in the HbA1c and fructosamine levels in diabetic animals treated with curcumin and/or aminoguanidine are related to the small decreases in the glycemia promoted by these treatments. Corroborating our findings, Chougala et al. [33] also noticed discrete effects on glycemia when STZ-diabetic rats were fed for 16 weeks with curcumin or aminoguanidine supplemented diets. On the other hand, it must be highlighted that the effects on AGE levels (biomarkers of advanced glycation) in the plasma, liver, and kidneys of diabetic animals were similar among all treatments (insulin, curcumin, and aminoguanidine), suggesting that later glycation events were inhibited to the same extent by the different treatments; however, curcumin and aminoguanidine act through mechanisms potentially different from those of insulin. Besides, the treatment of diabetic rats with insulin also increased the levels of components that detoxify AGEs and the activities of antioxidant enzymes, while decreasing the biomarkers of glycoxidative damage, including TBARS, PCO, and AGEs. However, considering that insulin effectively controlled the glycemia of STZ-induced diabetic rats since the beginning of the experiment, it seems reasonable to assume that insulin prevented the impairments to the cytoprotective machinery and thus avoided the onset of glycoxidative stress in diabetic rats. Finally, treatment of diabetic rats with insulin also prevented body weight loss and proteinuria, decreased polyphagia, avoided dyslipidemia, and significantly decreased hepatic damage biomarkers.

Despite the benefits of treatment with 4 U/day insulin, it is known that chronic insulinization increases the risk of hypoglycemia [34]. Long-term insulinization has also been involved with the establishment of insulin resistance [35], thus requiring a progressive increase in the insulin dose to achieve the antihyperglycemic response, increasing the risk of hypoglycemia. Therefore, therapies combining insulin with natural bioactive compounds have been recently proposed as strategies to reach an effective glycemic control and attenuate the long-term diabetic complications related to glycoxidative stress, as well as avoid the adverse effects of long-term insulinization [9, 36, 37]. Recently, we investigated the effectiveness of a combined therapy between curcumin (90 mg/kg) and insulin in a minor dose (1 U/day) for glycemia management of STZ-diabetic rats, as well as its effects on oxidative stress in the liver and biomarkers of hepatic damage. Previously, Gutierres et al. [8] observed that the treatment of diabetic rats with curcumin+1 U/day insulin induced the same benefits as the treatment with 4 U/day insulin, since it increased hepatic antioxidant defenses, decreased oxidative damage markers, and lowered the plasma levels of ALT, AST, and ALP. However, it should be noted that, although curcumin+1 U/day insulin caused greater decreases in the glycemia than those of the isolated treatments, these levels have not been fully normalized, suggesting that diabetic animals treated with this combined therapy may still be vulnerable to the deleterious effects of glycation processes. Considering the findings of Gutierres et al. [8], the use of the combination of 90 mg/kg curcumin and aminoguanidine with insulin in a minor dose appears as a potentially applicable therapeutic strategy to avoid hypoglycemia, as well as being capable to mitigate or even avoid the diabetic complications related to glycoxidative stress.

The effects of curcumin on glycemia may be related to its ability for stimulating the release of insulin from pancreatic beta cells [38]. However, considering the massive destruction of pancreatic beta cells caused by STZ, other *in vivo* effects of curcumin probably exert more impact on its ability to mitigate glycemia in STZ-induced diabetic rats. It has been observed that curcumin is able to increase peripheral insulin sensitivity; the treatment of STZ-diabetic rats with curcumin-enriched yoghurt increased both peripheral insulin sensitivity and glucose tolerance, which seem to be associated with an increase in the AKT phosphorylation levels and GLUT 4 translocation in skeletal muscles [39]. However, since STZ-diabetic animals have severe diabetes with very high blood glucose levels, the antihyperglycemic effects of curcumin were probably subtle; this could also be true for the other effects of curcumin on the early markers of glycation, body weight gain, food intake, lipid profile, and biomarkers of hepatic (ALT, AST, and ALP) and renal (proteinuria) damage.

The effects of aminoguanidine (50 or 100 mg/kg) on decreasing glycemia in STZ-induced diabetic rats were also mild and may be related to their suppressive effects on AGEs generation. It is well-known that the AGE/RAGE axis triggers intracellular signaling pathways, causing oxidative stress and inflammation, which are directly associated with insulin resistance [40]. In addition, methylglyoxal (which is scavenged by aminoguanidine) seems to impair insulin signaling by suppressing stimulatory phosphorylation of the insulin receptor substrate 1 (IRS-1) and inhibiting the activity of phosphatidylinositol-3-kinase (PI3K) [41]. By enhancing insulin sensitivity, aminoguanidine also contributes to the slight improvements observed in the lipid profile and the biomarkers of hepatic damage. On the other hand, treatments with aminoguanidine alone, at both doses, were not effective in controlling body weight gain, food intake, and proteinuria in diabetic rats. Regarding the effects of aminoguanidine on renal integrity, literature reports divergent findings. Lv et al. [42] observed that the treatment of diabetic rats with aminoguanidine for 56 days reduced proteinuria. Conversely, Wilkinson-Berka et al. [43] found that the treatment of STZ-diabetic rats with aminoguanidine for 84 days did not reduce albuminuria. For these biomarkers, the combined therapy added beneficial effects to the aminoguanidine, since the association between curcumin and aminoguanidine, especially at the lower dose, maintained the benefits of curcumin in improving the body weight gain, food intake, and decreasing proteinuria.

Notably, the treatment of diabetic rats with 100 mg/kg aminoguanidine, alone or in combination with curcumin, promoted beneficial effects on various parameters in a similar way to the treatments with curcumin or with 50 mg/kg aminoguanidine, alone or combined. However, the higher aminoguanidine dose was neither able to avoid the increase in plasma ALP and protein urinary levels nor able to improve the activities of CAT (liver) and SOD (liver and kidneys). Moreover, curcumin and 100 mg/kg aminoguanidine treatment nullified the effects of curcumin on mitigating the damage to the liver and kidneys, since DCA-100 rats had increases in both plasma ALP and proteinuria levels. Considering these unfavorable effects, it can be assumed that high aminoguanidine dose, alone or combined with curcumin, may present some toxicity as a consequence of the high dose and/or its antagonism to the pharmacokinetics/pharmacodynamics of curcumin. Previous studies of our laboratory have also reported the unfavorable impacts of some combined therapies of bioactive molecules in *in vivo* models of metabolic disturbances. Arcaro et al. [44] found that the antidiabetic and antioxidant activities of curcumin were lost when administered in combination with piperine; moreover, this combined therapy was apparently toxic to the liver, since it increased the levels of ALT in the plasma of diabetic rats. Costa et al. [27] observed that the treatment of high-fat diet-fed mice with curcumin in combination with trigonelline impaired many of the benefits previously achieved by the treatments with these natural bioactive substances alone. Altogether, these studies corroborate our present findings on the loss of effectiveness of both curcumin and low-dose aminoguanidine on diabetes when the latter was coadministered at a high dose. In terms of future applicability, combinations of curcumin with aminoguanidine or other anti-AGE agents should take into account the dose of these bioactive molecules in order to avoid their dose-related or negative synergic toxic effects.

Another interesting finding of this study was the decrease in hepatic TBARS levels in diabetic rats, parallel to increased activities of the enzymes belonging to the glutathione redox system, GSH-Px and GSH-Rd. It can be hypothesized that, with the onset of the oxidative stress in DM, there was an increased production of lipid peroxides; those combined with lesser levels of GSH (in this study represented by NPSH groups) could induce compensatory mechanisms leading to increases in the GSH-Px activity and/or expression, as this enzyme has lipid peroxides as substrates [45]. Furthermore, glutathione S-transferase (GST) also participates in the detoxification of LPO products in the liver [46]. Altogether, it can be argued that lipid peroxides (estimated by TBARS) are overproduced in DM and could be more efficiently eliminated by increasing the levels of GSH-Px, GSH-Rd, or even GST. The low levels of NPSH in the liver of DYOG rats suggested increased oxidation of GSH, probably due to the increase of GSH-Px activity. However, it can be noted that this compensatory mechanism was not able to avoid the oxidative stress in the liver, in view of the increase in the PCO levels and the fall in the activities of SOD and CAT. Finally, diabetic rats also had increases in the activities of GSH-Px and GSH-Rd in the kidneys. However, unlike in the liver, the renal levels of TBARS were increased, suggesting that the production of lipid peroxides in the kidneys is higher than that in the liver. The low activity of GST in the kidneys compared with that in the liver may also explain the increases in the renal TBARS levels [46]. Thus, despite an increase in the activities of GSH-Px and GSH-Rd in the kidneys of diabetic rats, LPO continues to prevail in this tissue. However, it is important to mention that first, all treatments conducted in this study promoted responses similar to that of insulin, about the activities of GSH-Px and GSH-Rd in the liver and kidneys, and second, treated diabetic animals had changes in the glutathione system parameters that were identical to those of normal rats.

Our study also identifies novel beneficial mechanisms of curcumin and aminoguanidine action on lessening the glycoxidative stress of diabetic rats. Curcumin or aminoguanidine alone, mainly at the dose of 50 mg/kg, increased the activities of the antioxidant enzymes SOD (kidneys) and CAT (liver and kidneys) and the expression of components related to AGE detoxification, including AGE-R1 (liver and kidneys) and GLO 1 (liver). Thus, even without having a prominent effect on decreasing glycemia, both curcumin and aminoguanidine caused significant decreases in PCO and AGEs levels in the liver and kidneys of STZ-diabetic rats, probably due to their effects on these cytoprotective components. Increases in the activities of antioxidant enzymes due to the treatment with curcumin [7, 8] or with aminoguanidine [47, 48] have been reported in previous studies. It has also been noted that curcumin is able to increase the levels of both GLO 1 in the liver of high-fat diet-fed mice [27] and AGE-R1 in *ex vivo* models [49, 50]. Corroborating our findings about aminoguanidine effects on the increase in the components of AGE detoxification components, Rai et al. [51] found that aminoguanidine treatment of rats that consumed a high-fructose solution increased the AGE-R1 expression in *gastrocnemius* skeletal muscles and decreased the levels of AGEs in tissues. Lastly, to the best of our knowledge, this study shows the first evidence of aminoguanidine increasing GLO 1 expression in diabetic rats, which could be relevant for controlling AGE levels in the liver (both at 50 and 100 mg/kg) and kidneys (100 mg/kg), thus dampening glycoxidative stress.

The mitigation of glycoxidative stress in STZ-diabetic rats may find new therapeutic strategies based on the combination of curcumin and aminoguanidine; this seems viable because monotherapy inefficacies at improving either antioxidant defenses or AGE detoxification systems have been overcame by the combination between the two bioactive molecules. In the liver, in contrast with the isolated therapies, the combination of curcumin and 50 mg/kg aminoguanidine increased SOD activity. Curcumin+100 mg/kg aminoguanidine was also able to increase the CAT activity in the liver, which means the maintenance of the curcumin beneficial effects. In the kidneys, the combination of curcumin and 100 mg/kg aminoguanidine increased SOD activity, an effect not found with aminoguanidine alone. The combined therapy also increased renal GLO 1 levels, an effect not seen with 50 mg/kg aminoguanidine alone. Therefore, these findings reinforce the need for further studies with the combination of both curcumin and aminoguanidine and its potential to dampen the diabetic complications related to oxidative stress and advanced glycation events.

Additionally, the combination of curcumin and aminoguanidine promoted significant decreases in the plasma levels of triglycerides (DCA-50 and DCA-100) and total cholesterol (DCA-50), whose levels were lower than those of animals treated with curcumin or aminoguanidine alone and similar to the levels of NYOG rats. Thus, it can be suggested that these combined therapies were effective in preventing dyslipidemia of diabetic rats. In addition, the combined therapies added benefits to curcumin, since they were able to decrease NO_x_ levels, an effect attributed to the aminoguanidine. Aminoguanidine is a well-known selective iNOS inhibitor [52]. In DM, impaired cardiovascular function has been partially attributed to the pathological overexpression of iNOS in cardiovascular tissues [53]. Furthermore, high glucose levels are known to increase the synthesis of diacylglycerol, which is a potent activator of the protein kinase C (PKC); the activation of some PKC isoforms has been involved in the induction of iNOS in cardiovascular tissues, thus playing a critical role in the development of diabetic cardiovascular complications [54]. In light of this, our data provided evidence that curcumin-aminoguanidine combined therapy is effective in correcting the abnormalities of iNOS activity (indirectly estimated by NO_x_ levels), since the overproduction of NO_x_ in STZ-diabetic rats was prevented by the combined therapies. Finally, the decrease in the PON 1 activity in diabetic rats was prevented by the treatments with curcumin and aminoguanidine, alone or in combination. PON 1 circulates predominantly with HDL and has antioxidant activity by hydrolyzing lipid peroxides within lipoproteins [55]. In diabetes, decreases in the activity of PON 1 have been related to glycation processes [56]. Bansal et al. [57] found a negative correlation between serum AGE levels and PON 1 activity in individuals with type 2 DM; authors suggested that the AGE overproduction that occurred under hyperglycemic conditions may be affecting PON 1 activity. Therefore, the decrease of AGE levels promoted by the treatments with curcumin and aminoguanidine may be helpful to preserve the activity of PON 1 in diabetic rats. In short, our findings show that a combined curcumin-aminoguanidine therapy prevent dyslipidemia, increase PON 1 activity, and normalize NO_x_ levels in DM; this may constitute a potentially effective new strategy for the management of cardiovascular complications observed in DM.

## 5. Conclusion

The study of the underlying biochemical cascades that lead to advanced glycation and ROS generation in DM may provide new therapeutic strategies to attenuate or even prevent the progression of the diabetic complications. This study demonstrated that treating diabetic rats with curcumin and aminoguanidine, individually or in combination, promoted several benefits in the biomarkers of metabolic disturbances and glycoxidative stress in the liver and kidneys. The results achieved in this study provide novel insights about the *in vivo* mechanisms by which these bioactive compounds activate cytoprotective components to counteract the harmful impacts of the glycoxidative stress in diabetes, with emphasis on the endogenous antioxidant defenses and components participating in AGE detoxification. The beneficial effects of the combination between curcumin and aminoguanidine in diabetes reinforce the promising potential of natural and synthetic bioactive compounds with antioxidant and anti-AGE properties. If we consider the potential applicability of the current study, in the future, it will be important to further explore a combined administration of curcumin with aminoguanidine or with other anti-AGE agents; this could arise as a complementary therapeutic option to be used with antihyperglycemic agents for combating the diabetic complications.

## Figures and Tables

**Table 1:** Biochemical parameters of streptozotocin-induced diabetic rats after 45 days of treatment with yoghurt enriched with curcumin, alone or in combination with aminoguanidine.

	Groups							
	NYOG	DYOG	DINS	DC	DA-50	DCA-50	DA-100	DCA-100
HbA1c (%)	10.5 ± 0.4	18.6 ± 0.3^a^	13.8 ± 0.6^a,b^	15.3 ± 0.5^a,b,c^	17.1 ± 0.4^a,b,c^	17.0 ± 0.4^a,b,c^	16.0 ± 0.4^a,b,c^	15.6 ± 0.5^a,b,c^
Fructosamine (*μ*mol/L)	74.4 ± 6.3	209.9 ± 18.3^a^	118.9 ± 9.8^a,b^	168.5 ± 12.2^a,b,c^	163.6 ± 8.0^a,b,c^	172.6 ± 6.7^a,b,c^	159.6 ± 9.4^a,b,c^	167.9 ± 12.3^a,b,c^
Triglycerides (mg/dL)	94.4 ± 9.5	229.5 ± 23.3^a^	81.0 ± 10.1^b^	157.9 ± 18.9^a,b,c^	150.1 ± 22.3^a,b,c^	115.4 ± 9.7^b^	158.5 ± 11.9^a,b,c^	109.2 ± 9.9^b^
Total cholesterol (mg/dL)	57.0 ± 2.0	72.2 ± 1.7^a^	57.4 ± 2.0^b^	63.9 ± 1.1^a,b,c^	64.2 ± 2.2^a,b,c^	56.9 ± 1.9^b,d,e^	65.3 ± 2.4^a,b,c^	60.3 ± 2.9^b^
ALT (U/L)	80.9 ± 3.6	226.5 ± 13.5^a^	93.4 ± 6.4^b^	173.9 ± 13.6^a,b,c^	171.1 ± 12.1^a,b,c^	182.1 ± 21.9^a,b,c^	178.2 ± 14.7^a,b,c^	169.7 ± 9.0^a,b,c^
AST (U/L)	107.2 ± 4.5	182.8 ± 17.1^a^	96.9 ± 6.4^b^	137.1 ± 8.3^b^	134.2 ± 12.8^b^	139.0 ± 14.9^b^	135.7 ± 8.2^b^	130.5 ± 8.2^b^
ALP (U/L)	176.1 ± 10.4	1313.2 ± 93.8^a^	275.7 ± 30.45^b^	915.3 ± 69.15^a,b,c^	911.0 ± 90.02^a,b,c^	903.4 ± 89.80^a,b,c^	1354.3 ± 97.4^a,c,d,e,f^	1113.7 ± 90.1^a,c,g^
Proteinuria (mg/24 h)	5.4 ± 0.9	17.7 ± 1.4^a^	5.0 ± 0.8^b^	11.8 ± 0.9^a,b,c^	18.7 ± 0.9^a,c,d^	12.0 ± 1.2^a,b,c,e^	16.8 ± 1.3^a,c,d^	14.7 ± 1.2^a,c^

Values are expressed as the mean ± standard error of the mean (SEM) (*n* = 10). NYOG: normal rats treated with yoghurt; DYOG: diabetic rats treated with yoghurt; DINS: diabetic rats treated with 4 U/day insulin; DC: diabetic rats treated with 90 mg/kg curcumin in yoghurt; DA-50: diabetic rats treated with 50 mg/kg aminoguanidine in yoghurt; DCA-50: diabetic rats treated with 90 mg/kg curcumin+50 mg/kg aminoguanidine in yoghurt; DA-100: diabetic rats treated with 100 mg/kg aminoguanidine in yoghurt; DCA-100: diabetic rats treated with 90 mg/kg curcumin+100 mg/kg aminoguanidine in yoghurt. Differences between groups were considered significant at *p* < 0.05 and were analyzed using one-way ANOVA followed by the Student-Newman-Keuls test. ^a^ Differences to NYOG. ^b^ Differences to DYOG. ^c^ Differences to DINS. ^d^ Differences to DC.^e^ Differences to DA-50. ^f^ Differences to DCA-50. ^g^ Differences to DA-100.

## Data Availability

The authors confirm that the data supporting the findings of this study are available within the article.
